# Model selection and parameter estimation for dynamic epidemic models via iterated filtering: application to rotavirus in Germany

**DOI:** 10.1093/biostatistics/kxy057

**Published:** 2018-09-27

**Authors:** Theresa Stocks, Tom Britton, Michael Höhle

**Affiliations:** Department of Mathematics, Stockholm University, 10691 Stockholm, Sweden

**Keywords:** Iterated filtering, Model selection, Parameter inference, Partially observed Markov process, Rotavirus surveillance data, Seasonal age-stratified SIRS model

## Abstract

Despite the wide application of dynamic models in infectious disease epidemiology, the particular modeling of variability in the different model components is often subjective rather than the result of a thorough model selection process. This is in part because inference for a stochastic transmission model can be difficult since the likelihood is often intractable due to partial observability. In this work, we address the question of adequate inclusion of variability by demonstrating a systematic approach for model selection and parameter inference for dynamic epidemic models. For this, we perform inference for six partially observed Markov process models, which assume the same underlying transmission dynamics, but differ with respect to the amount of variability they allow for. The inference framework for the stochastic transmission models is provided by iterated filtering methods, which are readily implemented in the R package pomp by King *and others* (2016, Statistical inference for partially observed Markov processes via the R package pomp. *Journal of Statistical Software***69**, 1–43). We illustrate our approach on German rotavirus surveillance data from 2001 to 2008, discuss practical difficulties of the methods used and calculate a model based estimate for the basic reproduction number $R_0$ using these data.

## 1. Introduction

Infectious disease outbreaks are often observed in the form of uni- or multivariate time series, e.g. as the number of newly reported cases aggregated over some time period. In order to estimate the relationship between variables, a classical statistical approach is the use of time series analysis of the incident cases, e.g. as in Finkenstädt *and others* (2002), Held *and others* (2006). These models are useful for prediction and forecasting, however, they teach us little about the dynamics of the disease spread, which are crucial if one wants to, e.g. assess the risk of emerging pathogens or evaluate the impact of control measures such as vaccination. Hence, another popular approach are transmission models, i.e. dynamic models which reflect the mechanisms of disease spread between individuals explicitly (Anderson and May, 1991; Keeling and Rohani, 2008; Diekmann *and others,* 2013). However, despite their wide application in infectious disease epidemiology, the choice between a stochastic and a deterministic transmission model for a specific data set is often subjective and based on expert knowledge or non-generalizable, ad hoc preferences (Sun *and others,* 2015). Additionally, in case of a stochastic transmission model the corresponding likelihood function is, except for the simplest cases, intractable. This makes inference for this type of models complicated which in turn makes a thorough analysis for model selection and parameter inference difficult. As it becomes more common to include the results of dynamic models as part of data analysis in infectious disease epidemiology, this challenge becomes increasingly interesting for, for example, public health authorities. Hence, the objective of this article is to demonstrate the application of an inference framework which facilitates *(i) model selection for both, deterministic and stochastic transmission models* and *(ii) parameter inference and quantification of the uncertainties of the estimates based on the observed data*. The idea of our work is similar to the one in Sun *and others* (2015), however, the inference method and area of application are different: we use iterated filtering methods to perform inference and demonstrate our approach on an age-structured model for rotavirus transmission in Germany. In our work, we consider a simplified version of the SIRS-type model used in Weidemann *and others* (2013) who analyzed the same data in a different context, including fewer disease states and fewer age classes but accounting for seasonality, age-structure and, in particular, more variability.

A convenient framework to answer infectious disease epidemiology related questions in a model based and data driven way are partially observed Markov processes (POMP). This model class consists of two model components: (i) an unobserved Markovian transmission process, which can be either continuous or discrete in time and in our application operates on the population level describing the dynamics of disease spread and (ii) an observation model, which relates the data collected at discrete points in time to the transmission model. In our work, we compare three different transmission models, which all assume the same underlying disease mechanisms but differ with respect to the amount of stochasticity they allow for. Namely, we compare a set of ordinary differential equations (ODEs), a continuous time Markov chain (CTMC), which is purely driven by demographic stochasticity, and an overdispersed CTMC which allows for variability beyond demographic noise by stochastic transmission rates (Bretó *and others,* 2009; He *and others,* 2009). All three approaches have their advantages and shortcomings. Models based on a deterministic transmission model component are preferably used when the population size is large and since they assume no randomness in the transmission, analysis and inference is easier and hence very popular among practitioners. However, these models explain any discrepancy between the deterministic model and the data by observation noise since this is the only element where stochasticity enters. In observed data in turn, we see fluctuations, which are clearly not due to variation in data collection alone but, e.g. due to environmental changes, different genotypes or variability in social behavior such as super spreaders etc. Most of these possible sources of variability are in part not sufficiently understood or not measurable in any way. Hence, stochastic variation in the transmission model is potentially an essential element to capture in order to quantify these influences. However, inference is more complicated than in the deterministic case, because the likelihood is often a complex and high dimensional integral resulting from integrating out stochasticity or missing state information.

With increasing computational power, simulation-based methods to solve those high dimensional integrals gained more and more attention. In this work, we use iterated filtering (Ionides *and others,* 2015) for maximum likelihood estimation in partially observed epidemic models, which takes advantage of the fact that it is relatively easy to generate samples from the Markov process compared with evaluating its transition probabilities. The method is, among others, conveniently implemented in the R package pomp (King *and others,* 2016), which spans a wide collection of inference tools for both deterministic and stochastic underlying transmission models. Other likelihood- and simulation-based inference methods for POMP models are simulated moments (Kendall *and others,* 1999), nonlinear forecasting (Sugihara and May, 1990), synthetic likelihood (Wood, 2010), or Bayesian approaches such as approximate Bayesian computations (Toni *and others,* 2009) and particle MCMC (Andrieu *and others,* 2010).

Since the package was developed fairly recently, we also comment on our experiences with the package and discuss its scientific as well as practical use. We base our criteria for model selection on Akaike Information Criterion (AIC) and coverage, i.e. how often the data is covered by the 95% point wise in-sample prediction interval evaluated at the best choice of parameters. The reasoning behind this choice is that we are interested in a relative measure assessing the trade-off between goodness-of-fit and complexity of the models on the one hand, and in an absolute measure of how well the models actually deal with variability in the data on the other hand. Our analysis will show for the particular data set at hand, to obtain the lowest AIC and best coverage it is important to include variability in the form of overdispersion into both model components. In Section 2, we give a short overview of rotavirus disease and the German rotavirus surveillance data. In Section 3, we present three different but related transmission models for the data. We explain how the transmission models are connected to the data via an observation component and formulate an expression for the basic reproduction number $R_0$. In Section 4, we present the inference framework and give some details concerning implementation. We perform a simulation study to demonstrate the suitability of the inference method for each of the models in Section 5. In Section 6, we apply our methods to data introduced in 2 and compare the findings to the approach used in Weidemann *and others* (2013). Section 7 ends the article with a discussion.

## 2. Motivating example: rotavirus disease

**Fig. 1.**
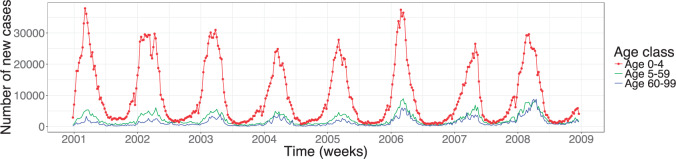
Weekly number of new rotavirus cases among the three aggregated age groups of 0–4, 5–59, and 60–99 years of age in Germany between 2001–2008. The values we used for scaling up are the median estimates of the under-reporting parameter from the averaged posterior distribution inferred in Weidemann *and others* (2013), namely 4.3% in the former western federal states (WFS) and 19.0% in the former eastern federal states (EFS) between 2001 and 2004 and 6.3% in WFS and 24.1% in EFS from 2005 onward. This sudden increase in reporting behavior is due to a nationwide change in the reimbursement of hospitalized rotavirus cases from January 2005.

Rotavirus disease is a childhood disease and the primary cause for gastroenteritis in infants and young children, while adults are rarely infected (Grimmwood and Lambert, 2009). By the age of five nearly every child has been infected with the virus at least once (CDC, 2017). The virus spreads by direct transmission on the faecal oral route. The incubation time of rotavirus is around 2 days, while severe symptoms last for approximately 4–8 days (CDC, 2017). Reinfection is possible because neither natural infection with rotavirus nor rotavirus vaccination provides full protection from future infections. The data, we analyze is the weekly reported number of new, laboratory-confirmed rotavirus cases among children younger than 5 years of age, individuals between age 5–59 and elderly aged 60–99 years from 2001 to 2008 in Germany. After 2008, a significant impact on the rotavirus incidence by the increasing vaccination coverage is observed (Weidemann *and others,* 2013). The data are available through SurvStat@RKI (RKI, 2017) and were previously analyzed in Weidemann *and others* (2013) as part of advising the German standing board of vaccination (STIKO) about the possible impact of a recommendation of rotavirus vaccination for children. Such modeling questions are typical in a public health context—mathematical modeling with SIRS-type models of rotavirus has already been carried out for different countries, cf. e.g. Atkins *and others* (2012); Weidemann *and others* (2013); Martinez *and others* (2016) and references therein. One problem with the available routine surveillance data is under-reporting, however, since our focus is on methodological insights, we use the results from Weidemann *and others* (2013) and simply scale up the available data with the inferred under-reporting factors. The time series depicted in Figure 1 shows the scaled up data for the three age groups. The case report data clearly shows seasonal variation of rotavirus.

## 3. Methods

We formulate three different transmission models, which assume the same transmission mechanisms but differ with respect to the amount of variability they allow for. The three different models are (i) a CTMC with demographic noise, (ii) an overdispersed CTMC with demographic noise and stochastic transmission rates, and (iii) a deterministic transmission model. Additionally, we investigate the role of overdispersion in the observation model, leading to two distinct models one of which allows for overdispersion and one that does not. By combining the three transmission models with the two observation models we obtain six different models for rotavirus infection, cf. Table A.1 in the supplementary material available at *Biostatistics* online, where Model DtSt contains the least stochasticity and Model St+St+ the most. We finish this section by deriving an expression for the basic reproduction number $R_0$ based on the transmission dynamics assumed here.

### 3.1. Transmission models

#### 3.1.1. CTMC with demographic noise.

**Fig. 2.**
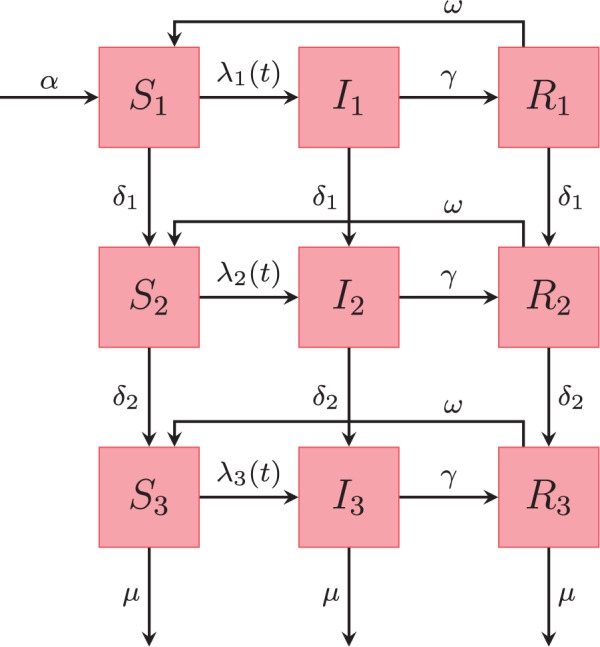
Schematic representation of the states in our SIRS model with three age classes. The rates on the arrows are explained in the text. Table 1 provides a list of notation, specifying which parameters are estimated in this work, and which are assumed known from the literature.

We assume that the transmission model is a Markov process where individuals move between compartments at random times, as described in detail below. The schematic representation of our model is given in the flow diagram of Figure 2. We consider a model that subdivides the population into three age classes, i.e. age 0–4, age 5–59, and age 60–99 years, identified by the indices 1, 2, and 3, respectively. We choose this age stratification, because the disease burden is highest for young children, very low for children over 5 years of age and rises again later in life. The variables $S_k(t), I_k(t), R_k(t)\in \mathbb{N}$ with $k\in \{1,2,3\}$ count the number of susceptible, infectious, and recovered in each of the three age groups at time $t\in \mathbb{R}^+$. Concerning the rates, young children “age” with a rate $\delta_1$ into the second age class and individuals of age 5–59 “age” with a rate $\delta_2$ into the class of elderly (age 60 +). The vector $\boldsymbol{\lambda}(t)=(\lambda_{{1}}(t), \lambda_{{2}}(t),\lambda_{{3}}(t))'$ is called the *force of infection* and is the per capita rate at which susceptible individuals get infected, which depends on the number of currently infected individuals $I_k(t)$. Susceptible individuals can become infected by transmission from an infectious individual in one of the three age groups. The recovery rate $\gamma\in \mathbb{R}^+$ is assumed to be independent of age. We also assume that waning of immunity is independent of age and immunity from infection lasts for a limited, exponentially distributed period with mean $1/\omega \in \mathbb{R}^+$ after which the individual is again fully susceptible; for a discussion of those assumptions cf. Section 7. To account for demographics, we assume that the average population size, $N$, is constant, which implies that the birth rate, $\alpha N\in \mathbb{R}^+$, equals the overall death rate. Furthermore, it is assumed that death can only occur in the last age class with rate $\mu\in \mathbb{R}^+$ independent of the health status of the individual. This seems reasonable because in developed countries 90 % of the mortality comes from individuals older than 60 years, hence, premature death can be ignored for our purposes (Atkins *and others,* 2012). In the following, the notation is adopted from King *and others* (2016). We let $N_{AB}(t), t\geq 0$ denote a stochastic counting process counting the number of individuals which have moved from compartment A to compartment B during the time interval $[0,t)$ with $A, B \in \mathcal{X}$, where $\mathcal{X}=\{S_1, S_2, S_3, I_1, I_2, I_3, R_1, R_2,$$ R_3\}$ contains all compartments of our model. Furthermore, $N_{\centerdot A}(t)$ counts the number of births and $N_{A \centerdot}(t)$ counts the number of deaths in the respective compartment up until time $t$. The infinitesimal increment probabilities of a jump between compartments connected by an arrow (Figure 2) fully specify the continuous time Markov process describing disease transmission. Let $\Delta N_{AB}(t)=N_{AB}(t+\tau)-N_{AB}(t)$ count the number of individuals changing compartment in an infinitesimal time interval $\tau>0$. Then we define for the model depicted in Figure 2 the following system of transition probabilities:

(3.1) \begin{align*}\mathbb{P}[\Delta N_{\centerdot S_1}(t)=1 |\mathcal{F}_t]&= \alpha N \tau +o(\tau) \nonumber \\\mathbb{P}[\Delta N_{S_kS_{k+1}}(t)=1 |\mathcal{F}_t]&= \delta_k S_k(t)\tau+o(\tau)\quad \text{with $k\in \{1,2\}$} \nonumber \\ \mathbb{P}[\Delta N_{S_kI_k}(t)=1 |\mathcal{F}_t]&= \lambda_k(t) S_k(t)\tau +o(\tau) \quad \text{with $k\in \{1,2,3\}$} \nonumber \\ \mathbb{P}[\Delta N_{I_kI_{k+1}}(t)=1 |\mathcal{F}_t]&= \delta_k I_k(t)\tau +o(\tau)\quad \text{with $k\in \{1,2\}$}\nonumber \\ \mathbb{P}[\Delta N_{I_kR_k}(t)=1 |\mathcal{F}_t]&= \gamma I_k(t)\tau +o(\tau) \quad \text{with $k\in \{1,2,3\}$} \nonumber\\ \mathbb{P}[\Delta N_{R_kR_{k+1}}(t)=1 |\mathcal{F}_t]&= \delta_k R_k(t)\tau +o(\tau)\quad \text{with $k\in \{1,2\}$}\nonumber \\ \mathbb{P}[\Delta N_{R_kS_k}(t)=1 |\mathcal{F}_t]&= \omega R_k(t)\tau +o(\tau) \quad \text{with $k\in \{1,2,3\}$} \\ \mathbb{P}[\Delta N_{A \centerdot}(t)=1 |\mathcal{F}_t]&= \mu A(t)\tau +o(\tau) \quad \text{with $A\in \{S_3,I_3, R_3\}$} \nonumber\end{align*}

with the filtration $\mathcal{F}_t=\sigma (S_1(u),I_1(t),R_1(u),S_2(u),I_2(u),R_2(u),S_3(u),I_3(u),R_3(u), \forall$$0\leq u\leq t)$ denoting the history of the process until time $t$; cf. Appendix A.1 of the supplementary material available at *Biostatistics* online for how transition rates and the state variables are related. A consequence of this model formulation is that we assume that the waiting time of each individual within each compartment is exponentially distributed given the current state; we discuss how this assumption can be relaxed in Section 7. The number of newly infected individuals in age class $k$ accumulated in each observation time period $(t_{n-1},t_{n}]$ is then given as

(3.2) \begin{equation*}H_k^{\text{stoch}}(t_n)=N_{S_kI_k}(t_{n})-N_{S_kI_k}(t_{n-1}).\end{equation*}

The force of infection, written in vector notation, consists of the following components

(3.3) \begin{align*}\boldsymbol{\lambda}(t)&=\boldsymbol{\beta} \cdot\boldsymbol{I}(t)\cdot\kappa(t)\cdot \frac{1}{N},\end{align*}

where $\boldsymbol{I}(t)=(I_1(t),I_2(t),I_3(t))'$. Disease transmission is represented in a transmission-matrix $\boldsymbol{\beta}=(\beta_{kj})_{k,j\in\{1,2,3\}} $ where the parameter $\beta_{kj}$ denotes the average number of infectious contacts of infected individuals of age group $j$ with susceptible individuals of age group $k$ per time unit and hence is the product of contact rates and transmission probability. Our modeling should account for the possibility that individuals in each age class $k$ could have a different immune status due to age and hence having differing susceptibility to the disease, so we assume $\beta_{kj}=\beta_k$ for all $k,j \in \{1,2,3\}$. Furthermore, motivated by the rotavirus application, we introduce a seasonal periodic forcing to account for the clear seasonal pattern in the data. In contrast to $\boldsymbol{\lambda}(t)$ which is state dependent, the seasonal forcing function $\kappa(t)$ is assumed to be purely time dependent, i.e. it has the same effect in all age groups. We let

(3.4) \begin{equation*}\kappa(t)= \left(1+\rho\cos\left(\frac{2\pi}{w} t +\phi\right)\right),\end{equation*}

where $\rho\in [0,1] $ denotes the amplitude of the forcing, $2\pi/w \in \mathbb{R}^+$ denotes the period of the forcing (e.g. if the time unit is weeks, then $w=52$) and $\phi \in [0,2\pi]$ denotes the phase shift parameter. Note that with the choice of forcing in Equation (3.4) the parameter $\beta_{k} $ denotes the baseline transmission rate of an individual of age group $j$ with age group $k$ which varies between $(1-\rho)\beta_{k}$ and $(1+\rho)\beta_{k}$ during the year (Keeling and Rohani, 2008).

#### 3.1.2. Overdispersed CTMC with demographic noise and stochastic transmission rates.

Including sufficient stochasticity in a model as a way to capture drivers and phenomena not covered otherwise by the model (e.g. late season peak) is essential if one wants the model to fit the data, e.g. to explain the data well (Bretó *and others,* 2009). So far, we have accounted for stochasticity in the underlying system by assuming that individuals move between classes at random times. However, for the transmission model in Section 3.1.1 the role of randomness diminishes as the population size increases. The same occurs by modeling disease spread via stochastic differential equations (Fuchs, 2013). One way to introduce variability, which is independent of population size, is to assume stochastic fluctuations in the transmission rates. In this work, we follow the approach of Bretó *and others* (2009) and introduce a time continuous stochastic process, $\xi(t)$, which fluctuates around the value one and is multiplied onto the transmission rate. It can be shown that by choosing the corresponding integrated noise process, $\Gamma(t)$, in a way such that its increments are independent, stationary, non-negative, and unbiased the Markov property is retained (Bretó *and others,* 2009). One convenient example for a process which satisfies these conditions is a Lévy process with $\xi(t)=\frac{d}{dt}\Gamma(t)$, where marginally $ \Gamma(t+\tau )-\Gamma(t)\sim \text{Gamma}\left(\frac{\tau}{\sigma^2},\sigma^2\right)$ and where $\tau /\sigma^2$ denotes the shape and $\sigma^2$ the age-independent scale parameter with corresponding mean $\tau$ and variance $\tau\sigma^2$. Note that the integral of $\xi(t)$ over a time interval is well defined even though the sample paths of $\Gamma(t)$ are not formally differentiable (Bretó *and others,* 2009). We build this into the model as defined in Equation (3.1) and Equation (A.1) of the supplementary material available at *Biostatistics* online by modifying Equation (3.3) as

(3.5) \begin{equation*}\boldsymbol{\lambda}(t)=\boldsymbol{\beta}\frac{\boldsymbol{I(t)}}{N}\kappa(t)\xi(t).\end{equation*}

A consequence of this construction is that the variability of the distribution of the transmission process for a given time $t$ becomes larger compared with the case when there is no extra-demographic noise, hence we call the CTMC overdispersed. This form of overdispersion has been used in previous studies of infectious diseases, for example, measles (He *and others,* 2009), malaria (Bhadra *and others,* 2011), or rotavirus (Martinez *and others,* 2016).

#### 3.1.3. Deterministic transmission model.

Calculating the expected values of the equations in (A.1) in Appendix A.1 of the supplementary material available at *Biostatistics* online with the aid of (3.1), dividing by $\tau$, and taking the limit as $\tau \to 0$, we obtain the underlying deterministic form of the model

(3.6) \begin{align*}\frac{dS_k(t)}{dt} &= \alpha N \boldsymbol{1}_{\{k=1\}}+ \delta_{k-1} S_{k-1}(t)\boldsymbol{1}_{\{k=2,3\}}-\delta_k S_k(t)\boldsymbol{1}_{\{k=1,2\}}-\mu S_k(t)\boldsymbol{1}_{\{k=3\}}-\lambda_k(t) S_k(t)+ \omega R_k(t), \nonumber\\\frac{dI_k(t)}{dt} &= \lambda_k(t)S_k(t)+ \delta_{k-1} I_{k-1}(t)\boldsymbol{1}_{\{k=2,3\}}-\delta_k I_k(t)\boldsymbol{1}_{\{k=1,2\}}-\mu I_k(t)\boldsymbol{1}_{\{k=3\}}-\gamma I_k(t), \\\frac{dR_k(t)}{dt} &= \gamma I_k(t)+ \delta_{k-1} R_{k-1}(t)\boldsymbol{1}_{\{k=2,3\}}-\delta_k R_k(t)\boldsymbol{1}_{\{k=1,2\}}-\mu R_k(t)\boldsymbol{1}_{\{k=3\}}- \omega R_k(t),\nonumber\end{align*}

for $k=1,2,3$, the force of infection as in (3.3) and initial values satisfying $\sum_{i=1}^3 S_i(0)+I_i(0) +R_i(0)=N$. Note that for the deterministic model, the state variables are continuous rather than integer valued. The number of newly infected individuals in age class $k$ accumulated in each observation time unit $(t_{n-1},t_{n}]$, $ n \in \{1,2,\dots,\mathcal{N}\}$ is then given as

(3.7) \begin{equation*}H_k^{\text{det}}(t_n)=\int_{t_{n-1}}^{t_{n}} \lambda_k(t) S_k(t) dt.\end{equation*}

### 3.2. Observation models

To incorporate the count nature of the observations a natural first assumption would be to model the reported cases as realizations of a Poisson distributed random variable with a given time dependent mean to account for errors in classification of cases, including false positives. However, the data at hand suggests that the sample variance is larger than the sample mean, i.e. there is indication of overdispersion in the data. Another choice in this case is the negative binomial distribution which allows for additional variance. Hence, let the number of recorded cases $Y_{kn}, k\in \{1,2,3\}$ within a given reporting interval $(t_{n-1},t_{n}],$$ n \in \{1,2,\dots,\mathcal{N}\}$ be either

(3.8) \begin{equation*}Y_{kn} \sim \text{Pois}\left( H_k(t_n)\right)\quad \text{or} \quad Y_{kn} \sim \text{NegBin}\left( H_k(t_n), \frac{1}{\theta}\right)\!,\end{equation*}

with $H_k(t_n)$ being the true number of accumulated incidences in age class $k$ per time unit $(t_{n-1},t_{n}],$ in the model, cf. Equation (3.2) for the stochastic models and (3.7) for the deterministic model. Here $\text{NegBin}(\mu,1/\theta)$ denotes the negative binomial distribution with mean $\mu$ and variance $\mu+\theta\mu^2$. To reduce the number of parameters, the same dispersion parameter $\theta$ for all age classes is chosen as in Weidemann *and others* (2013).

### 3.3. Calculation of $R_0$

An important mathematical characteristic of an epidemic model is its basic reproduction number $R_0$; we apply the theory of Diekmann *and others* (2013), Chapter 7, in the following. $R_0$ is the expected number of new infections by a typical infectee during the early stage of an epidemic when everyone is susceptible. We can calculate the basic reproduction number $R_0$ for the deterministic transmission model which is, also, representative for the stochastic transmission model, because it approaches the deterministic system for a large population size $N$. Hence, let $\pi_k=N_k/N$ denote the community fraction of age class $ k\in \{1,2,3\}$ in the population and $\nu_k=1/(\gamma+\delta_k)$ for $k=1,2$ and $\nu_3=1/(\gamma+\mu)$ the average length an individual of type $ k\in \{1,2,3\}$ stays in the infectious compartment. With the seasonal forcing function chosen as in Equation (3.4) the expected number of $k$ individuals a $j$ individual infects if everyone is susceptible is $m_{kj}=\beta_{k}\pi_k \nu_j$, which represents a yearly average. The basic reproduction number $R_0 $ can then be calculated as the largest eigenvalue of the matrix $M=(m_{kj})_{k,j \in \{1,2,3\}}$. It follows with “proportionate mixing” that $R_0=\sum_{k=1}^3R_0^{(k)} \pi_k$ where we denote $R_0^{(k)}= \beta_k \nu_k$ as the age-specific basic reproduction number for age group $k=1,2,3.$ Since $R_0$ is a yearly average here, its interpretation as a threshold value for an epidemic changes slightly. Only if the yearly range does not cover the value one it can be interpreted in the traditional way.

## 4. Inference and implementation

We describe the general procedures of how to perform likelihood-based inference for a POMP for both a deterministic and a stochastic underlying transmission model. Since we use the R package pomp we give details on the implementation and provide the code at Stocks (2017).

### 4.1. Likelihood of a POMP

Let $\boldsymbol{Y}_n=(Y_{1n},\dots ,Y_{3n})'$ with $ n=1,\dots,\mathcal{N}$ denote the random variables counting the new cases at time $t_n$ in each of the three age-classes. These depend on the state of the continuous time transmission process $\boldsymbol{X}_n=(\boldsymbol{S}(t_n),\boldsymbol{I}(t_n), \boldsymbol{R}(t_n))$ at that time where, e.g. $\boldsymbol{S}(t_n)=(S_1(t_n),S_2(t_n),S_3(t_3))'$. Furthermore, we let $\boldsymbol{X}_{0:\mathcal{N}}=(\boldsymbol{X}_0,\dots,\boldsymbol{X}_\mathcal{N})$ and denote the parameter vector by $\boldsymbol{\psi}$. The joint density of the states and the observations is then defined as the product of the one-step transmission density, $f_{\boldsymbol{X}_n|\boldsymbol{X}_{n-1}}(\boldsymbol{x}_n|\boldsymbol{x}_{n-1};\boldsymbol{\psi})$, the observation density, $f_{\boldsymbol{Y}_n|\boldsymbol{X}_{n}}(\boldsymbol{y}_n|\boldsymbol{x}_{n};\boldsymbol{\psi})$, and the initial density $f_{\boldsymbol {X}_0}(\boldsymbol{x}_0;\boldsymbol{\psi})$ as

$$\begin{equation*}f_{\boldsymbol{X}_{0:\mathcal{N}},\boldsymbol{Y}_{1:\mathcal{N}}}(\boldsymbol{x}_{0:\mathcal{N}},\boldsymbol{y}_{1:\mathcal{N}};\boldsymbol{\psi})=f_{\boldsymbol{X}_0}(\boldsymbol{x}_0;\boldsymbol{\psi})\prod_{n=1}^{\mathcal{N}}f_{\boldsymbol{X}_n|\boldsymbol{X}_{n-1}}(\boldsymbol{x}_n|\boldsymbol{x}_{n-1};\boldsymbol{\psi})f_{\boldsymbol{Y}_n|\boldsymbol{X}_{n}}(\boldsymbol{y}_n|\boldsymbol{x}_{n};\boldsymbol{\psi}).\end{equation*}$$

The likelihood of the parameter vector is then given as the marginal density for a sequence of observations, $\boldsymbol{Y}_{1:\mathcal{N}}$, evaluated at the data, $\boldsymbol{y}^*_{1:\mathcal{N}}$, as

(4.1) \begin{align*}\mathcal{L}(\boldsymbol{\psi})&=f_{\boldsymbol{Y}_{1:\mathcal{N}}}(\boldsymbol{y}^*_{1:\mathcal{N}};\boldsymbol{\psi})=\int f_{\boldsymbol{X}_{0:\mathcal{N}},\boldsymbol{Y}_{1:\mathcal{N}}}(\boldsymbol{x}_{0:\mathcal{N}},\boldsymbol{y}^*_{1:\mathcal{N}};\boldsymbol{\psi})d\boldsymbol{x}_{0:\mathcal{N}},\end{align*}

see, e.g. King and Ionides (2017). Note that for our model this is a high-dimensional integral of dimension $(\mathcal{N}+1)\times 9$, which, except for the simplest cases, cannot be reduced analytically, see Section 4.1.2.

#### 4.1.1. Maximum likelihood estimation for deterministic transmission model.

Inference for partially observed dynamical systems with a deterministic underlying transmission model is relatively straightforward, because Equation (4.1) is computable. Indeed $\boldsymbol{X}_n=\boldsymbol{x}_n(\boldsymbol{\psi})$ is a known function of $\boldsymbol{\psi}$ for each $n$ if the initial values of the system are fixed and known, cf. Section 4.2. Hence, the integral over $\boldsymbol{X}_{0:\mathcal{N}}$ in Equation (4.1) is over a function which only has a single point mass, i.e. $\mathcal{L}(\boldsymbol{\psi})= f_{\boldsymbol{X}_{0:\mathcal{N}},\boldsymbol{Y}_{1:\mathcal{N}}}(\boldsymbol{x}_{0:\mathcal{N}}(\boldsymbol{\psi}),\boldsymbol{y}^*_{1:\mathcal{N}};\boldsymbol{\psi})$. The solution of the ODE system can be calculated numerically with, e.g. Runge–Kutta methods. Given this solution, maximum likelihood estimation boils down to a classical numerical optimization problem for a nonlinear function of the parameter vector. Maximum likelihood estimation for partially observed dynamical systems with a deterministic underlying transmission model is, e.g. implemented in the traj.match function in the R package pomp (King *and others,* 2016), which accesses R’s optim function. For further implementational details see Appendix B.1 of the supplementary material available at *Biostatistics* online. To determine a 95% confidence interval (CI) for the obtained estimates we calculate the profile log-likelihood for each parameter of interest and invert Wilks’ (likelihood ratio) test to get the desired intervals (Held and Sabanés Bové, 2013). To construct a 95% pointwise prediction interval for model realizations we calculate the 2.5% as well as the 97.5% quantile of the respective observation distribution (Equation 3.8) at each observation time $t_n$ with mean $H_k^{\text{det}}(t_n)$ (Equation 3.7) evaluated at the MLE and hence ignoring uncertainty in the parameters.

#### 4.1.2. Maximum likelihood estimation for stochastic transmission model.

For a partially observed stochastic transmission model the likelihood is not tractable, because knowledge of the parameters does not uniquely determine the solution of the transmission model and marginal likelihoods are very computationally demanding. Iterated filtering is a simulation-based method to find the maximum likelihood estimate, cf. Ionides *and others* (2015) and references therein. It explores the parameter space by adding noise to the parameters of interest and at each iteration approximates the likelihood of the perturbed model by evaluating the particle filter (Doucet *and others,* 2001). Particle filtering methods return a stochastic estimate of the log-likelihood marginalized over the latent states using re-sampling techniques which make them more efficient than standard Monte Carlo methods. The iterated filtering algorithm is implemented in the mif2 function in the R package pomp (King *and others,* 2016). All algorithm specific parameters are reported in Appendix B.2 of the supplementary material available at *Biostatistics* online. For an application oriented introduction to iterated filtering methods see Stocks, 2017. To obtain 95% CIs for the parameters we construct a Monte Carlo error adjusted profile log-likelihood (Ionides *and others,* 2017) of each parameter and use Wilks’ test. For each observation, a 95% prediction interval is computed based on 1000 realizations of the model evaluated at the MLE. Note that these are in-sample prediction intervals because all observations where used to fit the model.

### 4.2. Initial values and starting values

Optimally, all parameters of a model can be inferred from the data. However, our investigations showed that the inference algorithms are very sensitive to starting values of parameters as well as initial values of the system and fail if those values are chosen completely at random. We discuss in detail how to overcome this practical issue in Appendix C of the supplementary material available at *Biostatistics* online. Based on this analysis we estimate the two seasonality parameters $\phi,\rho$, the age-specific susceptibilities $ \beta_1, \beta_2$, and $ \beta_3$ and the overdispersion parameters $\sigma$ and $\theta$ in the following.

## 5. Simulation study

We perform a simulation study to demonstrate the suitability of the inference method for each of the models presented in Table A.1 of the supplementary material available at *Biostatistics* online. As proof of concept, we generate one realization of each model with parameters chosen as the periodic equilibrium of the deterministic model (cf. Appendix C of the supplementary material available at *Biostatistics* online), which we then treat as data to estimate parameters from. All other parameters are fixed at biological plausible values shown in Table 1. After the inference we compare the obtained estimates to the true parameters which serves as validation of our implementation, the results can be found in Table D.2 in the supplementary material available at *Biostatistics* online. For all models the estimated parameters are in good accordance with the true parameters. As an example, Figure D.1 in the supplementary material available at *Biostatistics* online shows the simulated data from Model DtSt$+$, together with pointwise 95% prediction intervals obtained from the model evaluated at the MLE for the solution of the ODE. To provide evidence that the results are also consistent for more than one model realization we carry out a small additional study, the results of which can be found in Appendix D of the supplementary material available at *Biostatistics* online, cf. Figures D.2 and D.3. In order to check if observation noise and transmission noise are distinguishable and how the estimation results change under model misspecification we carry out a robustness study. For this we generate one realization of each model and fit the obtained realizations to every model respectively. We find that both noise components $\theta$ and $\sigma$ are indeed distinguishable from each other and parameters are estimated correctly even under model misspecification. However, Models St+St and StSt give a substantial amount of filtering failures in every iteration when fitting model realizations from models with overdispersion, for an example, cf. Figure D.4 in the supplementary material available at *Biostatistics* online. A filtering failure occurs if at some observation time point all suggested particles are too unlikely, i.e. the probability of each particle given the observation is below a certain threshold which generally implies that the model and data are inconsistent (King and Ionides, 2017). Even when doubling the number of particles the problem persists. This means that those two models fit data from other models (hence under the type of model misspecification we investigated) very poorly. We, therefore, conclude that for inference for stochastic transmission models, overdispersion in the observation model is an important ingredient in order to get robust results also under model misspecification. As a consequence, we exclude those two models from our comparative analysis. To investigate the performance of model selection, we calculate the AIC for each model fit, cf. Table D.3 of the supplementary material available at *Biostatistics* online. We find that for each realization, the respective true model always has the lowest AIC which confirms the correctness of our methods. To ensure that the AIC is a reasonable choice for the problem at hand we investigate in a small simulation study tailored to our specific model setting (SIRS in endemic state) how well the AIC can detect potential misspecifications in the transmission model. We find that for this particular case the AIC can explicitly discriminate between forms that directly affect the transmission event, for details, cf. Section D.2 of the supplementary material available at *Biostatistics* online.

**Table 1.** Full list of notation and parameter values used

Parameter	Explanation	Value	Unit
$N$	Population size	$82 \,372 \,825$	individuals
$\alpha$	Individual birth rate	$1/(52\cdot78.9)$	week$^{-1}$
$\delta_1$	Aging from age group 1 (age 0–4) to 2 (age 5–59)	$1/(52\cdot 5)$	week$^{-1}$
$\delta_2$	Aging from age group 2 (age 5–59) to 3 (age 60+)	$1/(52\cdot 55)$	week$^{-1}$
$\mu$	Death rate (from age group 3 (age 60+) )	$1/(52\cdot 18.9)$	week$^{-1}$
$\beta_k$	Susceptibility of age group $k\in \{1,2,3\}$	To be estimated	individuals/week
$\omega$	Immunity waning rate	$1/(1\cdot 52)$	week$^{-1}$
$\gamma$	Recovery rate	$1$	week$^{-1}$
$\phi$	Phase shift of the seasonal forcing	To be estimated	1
$\rho$	Amplitude of the seasonal forcing	To be estimated	1
$\lambda_k(t)$	Force of infection of age group $k\in \{1,2,3\}$	Defined by Eqn. (3.3)	individuals/week
$\kappa(t)$	Seasonal forcing function	Defined by Eqn. (3.4)	1
$\theta$	Overdispersion parameter	To be estimated	1
$\sigma^2$	Scale parameter of the $\Gamma$-noise	To be estimated	1
$\tau$	Time step	To be estimated	week
$R_0$	Basic reproduction number	To be estimated	individuals

The inverse of the yearly birth rate equals the sum of the inverses of the yearly aging and death rates which add up to $78.9,$ which is the total life expectancy at birth in Germany averaged between the years 2001 and 2008, taken from The World Bank (2016). The averaged population size $N$ in Germany during that time period is taken from Statistisches Bundesamt (2016) and the reporting interval is 1 week. As in Atkins *and others* (2012), we assume that all individuals are immune against rotavirus for an average of one year. A more detailed discussion on the choice of this assumption can be found in Section 7.

## 6. Rotavirus infection application results

**Fig. 3.**
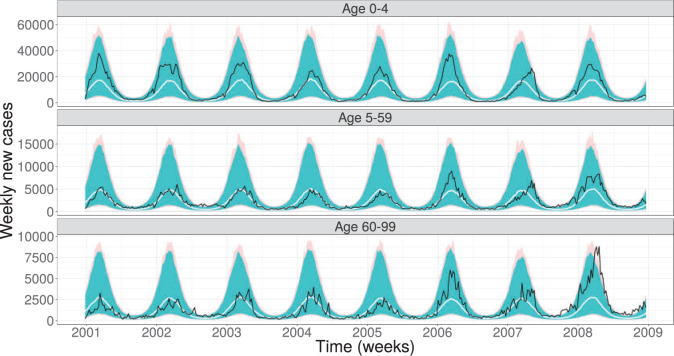
The 95% prediction interval (light shading) for 1000 realizations of Model St$+$St$+$ evaluated at the maximum likelihood estimate for the rotavirus incidence data (solid back line) and the median (solid white line). Furthermore, the 95 % prediction interval of these 1000 realizations for only the transmission model is shown (darker shading).

Parameter estimates, CIs as well as model diagnostics for the four models are given in Table 2. We report in the column “coverage” how often the actual data is covered by the pointwise 95% prediction interval of the respective model. For the stochastic transmission models StSt$+$ and St$+$St$+$ we also report how often the 95% prediction interval of the stochastic transmission model covers the data in order to investigate how much the observation noise additionally contributes to explaining the data. We find that matching Model DtSt to the data coincides well with the equilibrium results for the susceptibility parameters of the deterministic model without seasonality (cf. Equations C.5 in Appendix C.2 of the supplementary material available at *Biostatistics* online), although, this model obviously neither has an observation model nor a seasonal forcing component. However, the 95% prediction interval only covers 8.8% of the observed data and, hence, the model only poorly explains the variation in the data as is also reflected in a low log-likelihood value, cf. Figure E.5 in the supplementary material available at *Biostatistics* online. Fitting the deterministic model with overdispersion (Model DtSt$+$) to the data improves the fit by several thousands log units. The 95% prediction interval of the estimated parameter values covers now 96.2% of the data, cf. Figure E.6 in the supplementary material available at *Biostatistics* online. The difference between Model DtSt$+$ and Model St$+$St$+$ is the nature of their transmission model and we find that a stochastic transmission model improves the fit by additional 180 log units. We find that the coverage of the 95% prediction interval decreases to 90.6%, however, the coverage of the prediction interval of the transmission model is 28.9%, cf. Figure E.7 in the supplementary material available at *Biostatistics* online. The diagnostic plots which show how the log-likelihood of the mif2 model and the parameters evolve with each iteration can be found in Figures E.9 and E.11 in the supplementary material available at *Biostatistics* online. Finally, Model St$+$St$+$ allows for additional variability by having stochastic transmission rates. It has the highest log-likelihood and lowest AIC of the four models and is hence the best suited model to explain the data. The prediction interval covers 96.5% of the data. It is interesting to note is that now the transmission model by itself is able to explain nearly all the data because its prediction interval covers 93.7%, cf. Figure 3. This indicates that the observation noise component is not as strong as the three previous models suggest. A detailed interpretation of the estimates for $\beta_k$ and $R_0$ can be found in Section E.2 of the supplementary material available at *Biostatistics* online.

**Table 2.** Inference results for the four models with maximum likelihood estimate (MLE), 95% confidence intervals (CI), basic reproduction number $R_0$, 95% confidence interval for $R_0$, seasonal variability of $R_0$, coverage of the data by the 95% prediction interval of the full model, (coverage of the data 95% prediction interval only generated by the transmission model), log-likelihood (LL), Akaike information criterion (AIC), and standard error of the Monte Carlo approximations

**Model DtSt** (LL: $-$371 713, AIC: $743\,436$, coverage: 8.8%)							
	$\beta_1$	$\beta_2$	$\beta_3$	$\rho$	$\phi$		
MLE	12.659	0.237	0.418	0.151	0.037		
CI	[12.658, 12.659]	[0.237, 0.238]	[0.418, 0.419]	[0.151, 0.151]	[0.036, 0.038]		
$R_0$ = 1.065 (1.065–1.066), seasonal range: [0.904, 1.226]							
**Model DtSt$+$** (LL: $-$10 383.05, AIC: $20\,778.1$, coverage: 96.2%)							
	$\beta_1$	$\beta_2$	$\beta_3$	$\rho$	$\phi$	$\theta$	
MLE	11.718	0.284	0.477	0.129	0.067	0.221	
CI	[11.474, 11.956]	[0.270, 0.298]	[0.454, 0.503]	[0.125, 0.133]	[0.034, 0.100]	[0.205, 0.238]	
$R_0$ = 1.052 (1.021–1.083), seasonal range: [0.916, 1.188]							
**Model StSt$+$** (LL: $-10\,201.64$ (s.e. 1.94), AIC: $20\,415.28$, coverage: 90.6% [only transmission model: 28.9%])							
	$\beta_1$	$\beta_2$	$\beta_3$	$\rho$	$\phi$	$\theta$	
MLE	11.198	0.266	0.450	0.142	0.075	0.150	
CI	[10.889, 11.326]	[0.267, 0.289]	[0.435, 0.474]	[0.138, 0.146]	[0.040, 0.109]	[0.143, 0.164]	
$R_0$ = 1.000 (0.981–1.031), seasonal range: [0.859, 1.142]							
**Model St$+$St$+$** (LL: $-10\,060.19$ (s.e. 0.25), AIC: $20\,134.38$, coverage: 96.5% [only transmission model: 93.7%] )							
	$\beta_1$	$\beta_2$	$\beta_3$	$\rho$	$\phi$	$\theta$	$\sigma$
MLE	11.298	0.267	0.433	0.148	0.085	0.111	0.091
CI	[11.131, 11.531]	[0.259, 0.278]	[0.415, 0.449]	[0.136, 0.158]	[0.006, 0.165]	[0.114, 0.127]	[0.080, 0.106]
$R_0$ = 1.004 (0.986–1.027), seasonal range: [0.855, 1.152]							

### 6.1. Comparison with previous results

We find that Model St$+$St$+$ explains the data very well, although the transmission component does not go into the same epidemiological detail as, e.g. the work of Atkins *and others* (2012) or Weidemann *and others* (2013). Since we analyzed the same data as in Weidemann *and others* (2013) with the only difference that we directly scaled the data for under-reporting, it is very insightful to compare the model fits in order to see if it is worthwhile to model disease transmission very detailed and to give recommendations to future modelers of what we consider important. In Weidemann *and others* (2013) a Bayesian model averaging approach was used to infer the parameters of 18 very detailed but deterministic transmission models with negative binomial distributed observations. The models included 19 age classes, three susceptibility states, maternal antibody protection, distinguishing between symptomatic and asymptomatic cases resulting in an overall number of 266 age-specific states. Moreover, the data was split into two data sets (former eastern federal states and western federal states) so a region specific analysis was carried out and time specific birth and migration rates between age classes were included. In order to compare the fit of this approach with our model on equal grounds we choose the model with the highest weight (Model 18) evaluated at the posterior mode of the fitted parameters, available at Weidemann (2017). The AIC we obtain is 20 479.2, hence, based on AIC this very complex model is competitive with Model StSt+ but is outperformed by Model St+St+ by approximately 300 AIC units. Note, that this is a conservative estimate, for details concerning the calculations, see Section E of the supplementary material available at *Biostatistics* online. Assuming that all region specific under-reporting rates were one (no decomposition of the expected number of incidences into the two regions) we then sampled the reported incidences according to a negative binomial observation distribution and calculated the 2.5% and 97.5% sample quantiles. The plot of these prediction intervals and the rotavirus data used in our analysis is shown in Figure E.8 in the supplementary material available at *Biostatistics* online. A coverage of 95.9% was obtained which is comparable to Models DtSt+ and St+St+. We conclude that a model which is simpler with respect to clinical detail in the disease transmission is sufficient in terms of fit to explain the rotavirus data as long as it accounts for overdispersion in both model components, for a discussion on this cf. Section 7.

## 7. Discussion

In this work, we demonstrated how an inference framework via iterated filtering can be applied to perform model selection and parameter estimation for POMP models in a statistical systematic way for German routine surveillance rotavirus data. We focused on the discrepancy between common epidemiological practice, which uses ODE models for large populations and more modern stochastic approaches, where statistical inference quickly becomes complicated. Furthermore, we demonstrated some practical difficulties when fitting those epidemic models to data, a task which has become increasingly important in the public health context. Although our model was focusing on rotavirus transmission in Germany, it could easily be modified to explain other infectious diseases which have comparable transmission characteristics. Based on AIC and coverage we found that for the specific data set, a POMP model which accounts for overdispersion in both the transmission model and the observational component performed best and, moreover, that the two noise components can be disentangled; He *and others* (2009) reached similar conclusions when modeling measles. Alternative model selection approaches would have been possible—see Gibson *and others* (2018) for a comprehensive overview and, e.g. Streftaris and Gibson (2012) and Lau *and others* (2014) for applications of such criterion for individual based epidemic models. Note also that AIC based on the marginal likelihood can have difficulties (Greven and Kneib, 2010; Gibson *and others,* 2018). For our 80 mio individuals model with count data response due to interval-censored observations, predictive scoring rules (Held *and others,* 2017) could circumvent these problems, but at the cost of many additional computations. Because simulation studies showed that AIC worked sufficiently well for our model selection setting (see Section D.2 of the supplementary material available at *Biostatistics* online), we did not pursue such strategies further. Throughout, we used a frequentist approach rather than a Bayesian setting for fitting the models. Bayesian approaches can be a natural choice in order to translate existing domain specific knowledge into prior distributions on unknown model parameters. However, as already stated in Bhadra (2010) and Gibson *and others* (2018) the choice of inference framework should be a pragmatic one and dependent on whether one wants to impose this prior knowledge on unknown parameters or not. Caution should be exercised as those prior assumptions could obscure the fact that the models reach a complexity which makes individual parameters hardly identifiable and consequently the prior plays a crucial, but sometimes unintended, role. From a technical perspective, the Bayesian analog of iterated filtering is particle MCMC, the simplest and most commonly used algorithm of which is the particle marginal Metropolis Hastings algorithm (PMMH) (Andrieu *and others,* 2010) also implemented in the pomp package. The conceptual difference between iterated filtering and PMMH is that iterated filtering explores simultaneously the parameter and state space in one iteration, while in PMMH in each iteration only a single noisy likelihood approximation for a parameter vector drawn at random from the proposal distribution is used (Ionides *and others,* 2015). Hence, for problems where filtering is a substantial computational expense, PMMH shows to perform much slower than mif2. For details, how those two methods fare against each other see Bhadra, 2010; Ionides *and others,* 2015. Although there exist approaches to make Bayesian inference algorithms for the class of dynamic models we consider computationally more efficient, cf. e.g. Dureau *and others* (2013); Murray *and others* (2016), the pomp package provides a convenient and flexible infrastructure for efficient simulation and a large variety of inference methods for POMP models. Using such flexible general purpose epidemic modeling software is of great interest for public health decision makers, and we hope to see such stochastic approaches in routine use of, e.g., assessing vaccination programs in the future.

The reduced complexity of our model came at the cost of neglecting some potentially important clinical details of rotavirus transmission. We did not consider maternal immunity, a latent period, premature death or immigration and we assumed that the waning immunity as well as the reporting rates, recovery rates and overdispersion parameters are age independent. Furthermore, we assumed that the population mixes homogeneously and that waiting times are exponentially distributed. The main reason for those simplifications was that the observations only carry information about time of diagnosis aggregated over one week which makes the separation of latent and infectious periods not identifiable as well as they do not contain information on how long people have been infected or who infected whom if we at the same time want to estimate the age-specific susceptibility parameters. If more was known about, e.g. age-specific recovery rates, we could have fixed the rates at those literature informed values and perform model selection, however, this data does to our knowledge not exist. Also, it would have been possible to add a latent period of known distribution, but since this would have been only be a two days shift, we decided to ignore this for simplification. To relax the homogeneously mixing assumption we could have assumed a “known” contact structure matrix as can, e.g. be obtained from the POLYMOD study (Mossong *and others,* 2008) and estimate the age-specific proportionality factors to this matrix, however, not the contact matrix itself. One option to enhance the exponential distribution could have been to divide the waiting period for a change in a certain compartment into several stages, yielding a gamma distribution, hence, a distribution with a more pronounced mode which is often more realistic. However, since the focus of this work was the demonstration of an inference and model selection framework we were aiming at a simple model.

In general, model complexity depends on which questions one wants to answer, e.g. more age classes or infectious states might be needed, if one was to assess vaccination strategies with our model. Nevertheless, we see from this work that, in terms of fit, simplicity has its virtues: adequate modeling of variability, can be more useful than trying to get every biological detail right, because even without this detail our model still fits the data very well. This is an important message to convey in practical public health modeling, because many transmission models tend to include overly many biological details in order to gain medical acceptance.

## Supplementary Material

kxy057_Supplementary_Materials
