# Supplementary material for: Model selection and parameter estimation for dynamic epidemic models via iterated filtering: application to rotavirus in Germany
Source: Biostatistics. 2018 Sep 27;21(3):400–16. doi: 10.1093/biostatistics/kxy057 (PMC7307980; doi:10.1093/biostatistics/kxy057)
Supplement: kxy057_Supplementary_Materials [file kxy057_supplementary_materials.pdf]

Biostatistics (2018), **0**, 0, *pp.* 1–22
doi:10.1093/biostatistics/biostat˙SUPPL˙revision

Model selection and parameter estimation for dynamic epidemic models via iterated filtering: application to rotavirus in Germany Supplementary Material

THERESA STOCKS*, TOM BRITTON, MICHAEL HÖHLE

Department of Mathematics, Stockholm University, 10691 Stockholm, Sweden

theresa.stocks@math.su.se

APPENDIX

The Supplementary Materials contain the specification of the investigated models and mathematical details of the CTMC model in Section A, implementational details in Section B, a practical solution of how to choose initial values and starting values in Section C, details of the simulation studies in Section D and further inference results for the rotavirus data in Section E.

A. Investigated Models

A.1 *CTMC Model*

The transition rates are related with the state variables in the following way:

$$\Delta S_1(t) = \Delta N_{\cdot S_1}(t) - \Delta N_{S_1 S_2}(t) - \Delta N_{S_1 I_1}(t) + \Delta N_{R_1 S_1}(t)$$

$$\Delta I_1(t) = \Delta N_{S_1 I_1}(t) - \Delta N_{I_1 I_2}(t) - \Delta N_{I_1 R_1}(t)$$

*To whom correspondence should be addressed.

© The Author 2018. Published by Oxford University Press. All rights reserved. For permissions, please e-mail: journals.permissions@oup.com

Model name	Transmission model	Observation model
DtSt	deterministic	no overdispersion
DtSt+	deterministic	overdispersion
StSt	CTMC	no overdispersion
StSt+	CTMC	overdispersion
St+St	CTMC with overdispersion	no overdispersion
St+St+	CTMC with overdispersion	overdispersion

Table A.1. Specification of the investigated models, where we write CTMC for the model in Section 3.1.1, CTMC with overdispersion as in 3.1.2 and deterministic as in 3.1.3 . Note that the + in the model names accounts for overdispersion in the respective model component.

$$
\begin{aligned}
\Delta R_1(t) &= \Delta N_{I_1R_1}(t) - \Delta N_{R_1R_2}(t) - \Delta N_{R_1S_1}(t) \\
\Delta S_2(t) &= \Delta N_{S_1S_2}(t) - \Delta N_{S_2S_3}(t) - \Delta N_{S_2I_2}(t) \\
\Delta I_2(t) &= \Delta N_{S_2I_2}(t) + \Delta N_{I_1I_2}(t) - \Delta N_{I_2I_3}(t) - \Delta N_{I_2R_2}(t) \\
\Delta R_2(t) &= \Delta N_{I_2R_2}(t) + \Delta N_{R_1R_2}(t) - \Delta N_{R_2R_3}(t) - \Delta N_{R_2S_2}(t) \\
\Delta S_3(t) &= \Delta N_{S_2S_3}(t) + \Delta N_{R_3S_3}(t) - \Delta N_{S_3\cdot}(t) - \Delta N_{S_3I_3}(t) \\
\Delta I_3(t) &= \Delta N_{S_3I_3}(t) + \Delta N_{I_2I_3}(t) - \Delta N_{I_3\cdot}(t) - \Delta N_{I_3R_3}(t) \\
\Delta R_3(t) &= \Delta N_{I_3R_3}(t) + \Delta N_{R_2R_3}(t) - \Delta N_{R_3\cdot}(t) - \Delta N_{R_3S_3}(t)
\end{aligned}
\tag{A.1}
$$

B. Implementational details

In the following we give the implementational details for the two inference methods used, namely trajectory matching for the deterministic transmission models and iterated filtering for the stochastic transmission models. The code is made available in Stocks (2017).

B.1 *Deterministic transmission model*

As optimizing algorithm we choose the Nelder-Mead method (Nelder and Mead, 1965). In order to address the potential problem of local maxima in the optimization we use 100 randomly chosen parameter constellations as starting values for the fitting algorithm. These values are drawn

uniformly from a hypercube which covers the estimates for β_i, $i \in \{1, 2, 3\}$ obtained from the endemic equilibrium of the deterministic model, cf. Section C plus minus a range of about 15-70% around them. For the other parameters we are estimating $(\rho, \phi, \theta, \sigma)$ we use values which seemed sensible after simulating. All starting ranges for the parameters can be found in the code available in Stocks (2017). In general, if the inference procedure gives consistent results for starting values drawn at random from a hypercube this increases the chances that a global maximum has been found and a reliable global search has been performed (King and Ionides, 2017). One practical problem for the models at hand is that if the relative convergence tolerance in the Nelder-Mead algorithm is very small, estimation fails due to a degeneracy of the Nelder-Mead complex. To get around this, we choose the relative tolerance as small as possible so the algorithm does not fail and re-use the obtained estimates as starting values for a second run of the inference algorithm with the same tolerance. Surprisingly, the implementation of the algorithm then usually takes a few additional iterations. This way we make sure that the algorithm really converges. Based on the obtained maxima for all starting values we select the one with the highest likelihood as our maximum likelihood estimate (MLE).

B.2 *Stochastic transmission model*

We use the τ -leaping algorithm to generate realizations from the stochastic transmission models (Gillespie, 2001). In the iterated filtering algorithm as the iterations proceed the intensity of the perturbation is successively reduced ("cooling") and the log-likelihood of the perturbed model gradually approaches the log-likelihood of the model of interest. However, for a finite number of iteration steps, the log-likelihoods of the two models are not identical and a final particle filter evaluation of the `mif2` model output is necessary in order to obtain the Monte Carlo estimated log-likelihood of the MLE. For the fitting carried out in the following we use 20 starting values drawn uniformly from a hypercube chosen as in Section B.1 . To make calculations feasible

with respect to time we accept approximation errors of the underlying stochastic transmission process by choosing a rather large simulation time step size of 1/10. The question arose if this approximation error is causing parts of the transmission and observation noises. We carried out a sensitivity analysis for the simulation step size and decreased the step size to 1/40 weeks without finding a significant change of the noise parameters. Hence, we believe that the choice of the step size does not increase the transmission and observation noise noticeably. For all `mif2` searches we used `Nmif=300` iterations, `Np=5000` particles, a cooling of the perturbations of `cooling.fraction.50=0.5` and random walk standard deviations `rw.sd` which vary dependent on the parameter between 0.001 and 0.2. It turned out that there is no precise rule of how to choose the magnitude of the random walk perturbations which the parameters undergo in the iterated filtering algorithm. It took some trial and error to find reasonable values. For each of the 20 `mif2` outputs we run 10 particle filters, each with 1000 particles. From this we calculate the average log-likelihood by taking the logarithm of the mean of the estimated likelihoods and the standard error of the Monte Carlo approximation for every parameter set. Among the 20 particle filter outputs, we choose the parameter vector with the highest average log-likelihood as the maximum likelihood estimate. Overall, the iterated filtering algorithm for the Models StSt+ and St+St+ took approximately 11h respectively when working on a computer with 20 cores. Therefore, we can highly recommend working with a computer cluster and parallelized `R` code as done by us.

C. Initial values and staring values

To overcome the practical issue of how to choose initial values and starting values, we decided to break down the complexity of the problem by assuming that the system is in a "periodic" equilibrium which we are then able to calculate from the deterministic transmission model in (3.6). We consider this a reasonable assumption because the pattern in the data is very regular,

cf. Figure 1. For the inference procedures described in the main manuscript we fix the initial values $\boldsymbol{X}_0$ at this equilibrium as calculated in the following in Equations (C.2), (C.3) and (C.4) and initialize the system well ahead of the first observations such that simulations equilibrate before the first observation is made. Furthermore, we investigate which and how many parameters of the deterministic transmission model are, in addition to the initial values of the system, identifiable from the data at hand assuming periodic equilibrium and use the obtained estimates as staring values for our inference methods. This practical solution works very well in our case, however, we are aware that the assumption that the system is in an equilibrium which we calculate from a deterministic system is rather conservative and that it might well be that there is more information contained in the non-equilibrium or non-deterministic dynamics.

C.1 *Equilibrium of the deterministic transmission model without seasonality*

We note that the seasonal component in the data allows us to estimate the phase shift parameter ϕ and the amplitude of the forcing ρ, however, there is no closed form solution for those two parameters. What remains to be investigated is how many parameters can be estimated from the data without this seasonal component which corresponds to analyzing the system in the endemic state. For this purpose, we divide the ODE system in (3.6) by N so we obtain the fractions of the population being susceptible, infected and recovered, i.e. $s_k(t) = S_k(t)/N, i_k(t) = I_k(t)/N$ and $r_k(t) = R_k(t)/N, k \in \{1,2,3\}$ with the sum over all fractions adding up to one and write $\lambda_k(t) = \beta_k i(t)$ with $i(t) = \sum_{k=1}^{3} i_k(t)$. We are interested in the system at equilibrium, i.e. when $ds_k/dt = di_k/dt = dr_k/dt = 0$. Here, we work out the values of the variables which we denote by $\tilde{s}_k, \tilde{i}_k, \tilde{r}_k, k \in \{1,2,3\}$ (Keeling and Rohani, 2008).

In order to obtain an endemic state without seasonality we take for each age group the mean over time and treat the obtained values as our data Y_{kn} which is the number of newly reported rotavirus cases aggregated over reporting intervals $(t_{n-1}, t_n]$, $n \in \{1,\ldots,\mathcal{N}\}$ in age class $k \in \{1,2,3\}$. In

the endemic state $\tilde{s}_k, \tilde{i}_k, \tilde{r}_k, k \in \{1, 2, 3\}$ also the number of reported cases is in equilibrium, hence $Y_{kn} = Y_{kn+1}$ for all $n \in \{1, \dots, \mathcal{N}\}$. For $t_n - t_{n-1} = 1$ and assuming no observation noise, this translates mathematically to

$$Y_{kn} = \int_{t_{n-1}}^{t_n} \beta_k \tilde{i}\tilde{s}_k N du = \beta_k \tilde{i}\tilde{s}_k N, \tag{C.1}$$

with $\tilde{i}(t) = \sum_{k=1}^{3} \tilde{i}_k(t)$ and which is independent of the time point t_n. Hence, we will only write $Y_k := Y_{kn}$ in what follows. Plugging (C.1) into the transformed Equations (3.6) at equilibrium we obtain the age- dependent prevalence without seasonality at the endemic level as

$$\tilde{i}_1 = \frac{Y_1}{(\gamma + \delta_1)N}, \quad \tilde{i}_2 = \frac{Y_2 + \delta_1 \tilde{i}_1 N}{(\gamma + \delta_2)N}, \quad \tilde{i}_3 = \frac{Y_3 + \delta_2 \tilde{i}_2 N}{(\gamma + \mu)N}. \tag{C.2}$$

We conclude that the data thus can inform the three endemic states $\tilde{i}_k, k \in \{1, 2, 3\}$, if we assume that the demographic parameters for μ, δ_1, δ_2 as well as the recovery rate γ, are fixed and known. Furthermore, since in the deterministic model the total population size N as well as the population sizes $N_k = S_k(t) + I_k(t) + R_k(t)$ of age class k are constant, we know that

$$\tilde{s}_1 + \tilde{i}_1 + \tilde{r}_1 = \frac{N_1}{N} = \frac{\alpha}{\delta_1}, \quad \tilde{s}_2 + \tilde{i}_2 + \tilde{r}_2 = \frac{N_2}{N} = \frac{\alpha}{\delta_2}, \quad \tilde{s}_3 + \tilde{i}_3 + \tilde{r}_3 = \frac{N_3}{N} = \frac{\alpha}{\mu}. \tag{C.3}$$

These equalities follow from the fraction of the population in age class k being equal to the fraction of the average lifetime spent in each age class. We can therefore express $\tilde{r}_k$ in terms of $\tilde{s}_k$ and $\tilde{i}_k$, if we assume additionally that the birth rate parameter α is fixed and known. The variables that remain to be estimated are $\tilde{s}_1, \tilde{s}_2$ and $\tilde{s}_3$. It can now be seen that by further assuming the immunity waning rate ω to be fixed and known we can also estimate β_1, β_2 and β_3. For this, we equate the above system to zero and solve the equations. We obtain by analytical derivations

$$\begin{aligned} \tilde{s}_1 &= \frac{\alpha - (\gamma + \delta_1)\tilde{i}_1 + \omega(\frac{\alpha}{\delta_1} - \tilde{i}_1)}{\delta_1 + \omega}, & \beta_1 &= \frac{(\gamma + \delta_1)\tilde{i}_1}{\tilde{s}_1 \tilde{i}}, \\ \tilde{s}_2 &= \frac{\delta_1 \tilde{s}_1 - (\gamma + \delta_2)\tilde{i}_2 + \delta_1 \tilde{i}_1 + \omega(\frac{\alpha}{\delta_2} - \tilde{i}_2)}{\delta_2 + \omega}, & \beta_2 &= \frac{(\gamma + \delta_2)\tilde{i}_2 - \delta_1 \tilde{i}_1}{\tilde{s}_2 \tilde{i}}, \end{aligned} \tag{C.4}$$

$$\tilde{s}_3 = \frac{\delta_2\tilde{s}_2 - (\gamma+\mu)\tilde{\tilde{i}}_3 + \delta_2\tilde{\tilde{i}}_2 + \omega(\frac{\alpha}{\mu} - \tilde{\tilde{i}}_3)}{\mu+\omega}, \qquad \beta_3 = \frac{(\gamma+\mu)\tilde{\tilde{i}}_3 - \delta_2\tilde{\tilde{i}}_2}{\tilde{s}_3\tilde{\tilde{i}}}.$$

We thus conclude the following: given we observe the incidence of the three different age-groups, the deterministic system at equilibrium enables identifiability of the two seasonality parameters ϕ, ρ plus three more parameters, e.g. β_1, β_2 and β_3 assuming all other parameters are known.

C.2 *Equilibrium results for β_k*

As discussed above we estimate the susceptibility parameters β_k with $k \in \{1,2,3\}$ and the parameters for the seasonal forcing function ρ and ϕ from the available data. Moreover, we will estimate the overdispersion parameter θ for the Models DtSt+, StSt+ and St+St+ and additionally the shape parameter of the transmission noise σ^2 for Model St+St+. All other parameters are fixed at biological plausible values shown in Table 1. As a first inference step we calculate the β_k's from the equilibrium results. the obtained estimates for β_k as starting values for the other estimation procedures of all four models and the endemic state estimates as initial values for the transmission model.

In the rotavirus data the mean number of weekly reported new cases in age groups 1,2 and 3 between the years 2001 and 2008 are 10009, 2254 and 1364, respectively. Solving the calculations from Equation (C.4) we obtain

$$\hat{\beta}_1 = 12.66, \quad \hat{\beta}_2 = 0.24, \quad \hat{\beta}_3 = 0.42, \tag{C.5}$$

for the deterministic system.

D. Details Simulation study

The results of the simulation study in Section 5 can be found in Table D.2. As proof of concept Figure D.1 shows the model fit for a realization of Model StSt+ under the true model. To confirm that the results are also valid for more than one model realization we conducted a small additional

study for the most stochastic models of each model type, i.e. DtSt+ and St+St+. The results are shown in Figures D.2 and D.3 and show good accordance with the true parameter values used for simulation.

Model DtSt

	β_1	β_2	β_3	ρ	ϕ
TP	12.657	0.238	0.420	0.150	0.100
MLE	12.657	0.238	0.420	0.150	0.100
CI	[12.651,12.664]	[0.238,0.238]	[0.419,0.421]	[0.150,0.150]	[0.099,0.100]

Model DtSt+

	β_1	β_2	β_3	ρ	ϕ	θ
TP	12.657	0.238	0.420	0.150	0.100	0.500
MLE	12.658	0.235	0.429	0.156	0.135	0.473
CI	[12.316,12.998]	[0.219,0.253]	[0.398, 0.463]	[0.15,0.162]	[0.097,0.173]	[0.441,0.509]

Model StSt+

	β_1	β_2	β_3	ρ	ϕ	θ
TP	12.657	0.238	0.420	0.150	0.100	0.500
MLE	12.794	0.235	0.407	0.149	0.102	0.467
CI	[12.454, 13.106]	[0.221, 0.254]	[0.382, 0.440]	[0.144, 0.156]	[0.061, 0.143]	[0.437, 0.505]

Model St+St+

	β_1	β_2	β_3	ρ	ϕ	θ	σ
TP	12.657	0.238	0.420	0.150	0.100	0.300	0.050
MLE	12.390	0.248	0.431	0.152	0.113	0.313	0.034
CI	[12.053, 12.715]	[0.236, 0.266]	[0.408, 0.463]	[0.145, 0.160]	[0.071, 0.166]	[0.293, 0.343]	[0.019, 0.057]

Model St+St

	β_1	β_2	β_3	ρ	ϕ	σ
TP	12.657	0.238	0.420	0.150	0.100	0.050
MLE	12.697	0.240	0.422	0.147	0.140	0.047
CI	[12.672, 12.825]	[0.236, 0.242]	[0.421, 0.425]	[0.146, 0.158]	[0.033, 0.104]	[0.048, 0.052]

Model StSt

	β_1	β_2	β_3	ρ	ϕ
TP	12.657	0.238	0.420	0.150	0.100
MLE	12.670	0.238	0.420	0.150	0.104
CI	[12.645, 12.683]	[0.238, 0.239]	[0.419, 0.422]	[0.149, 0.151]	[0.094, 0.114]

Table D.2. Simulation study results for the models with true parameter (TP), maximum likelihood estimate (MLE) and calibration of the 95% confidence interval (CI).

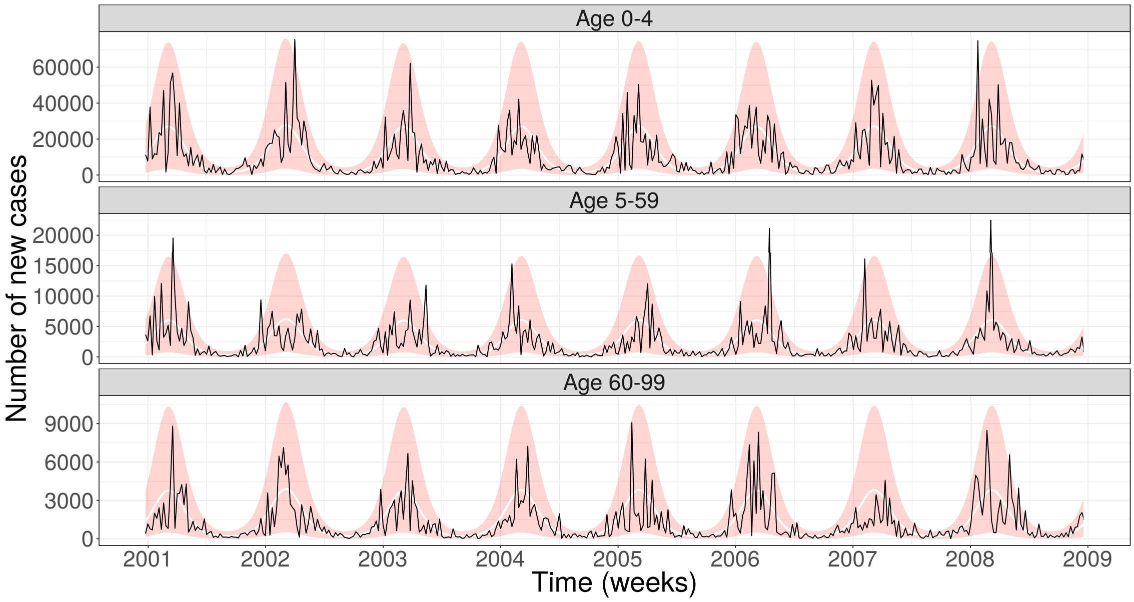

Fig. D.1. The 95% prediction interval (shading) for realizations of Model DtSt+ evaluated at the maximum likelihood estimate for simulated data (solid back line) and model mean (solid white line).

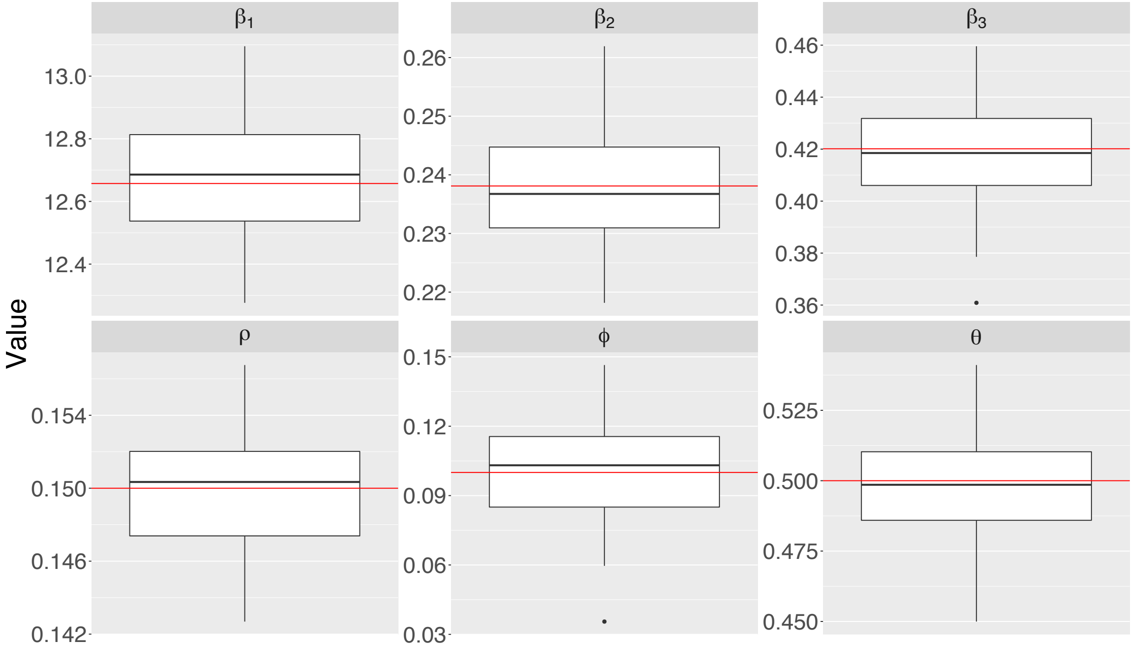

Fig. D.2. Boxplot of the MLEs inferred from 100 model realizations of Model DtSt+. The red horizontal lines are the true parameter values used to generate the simulations.

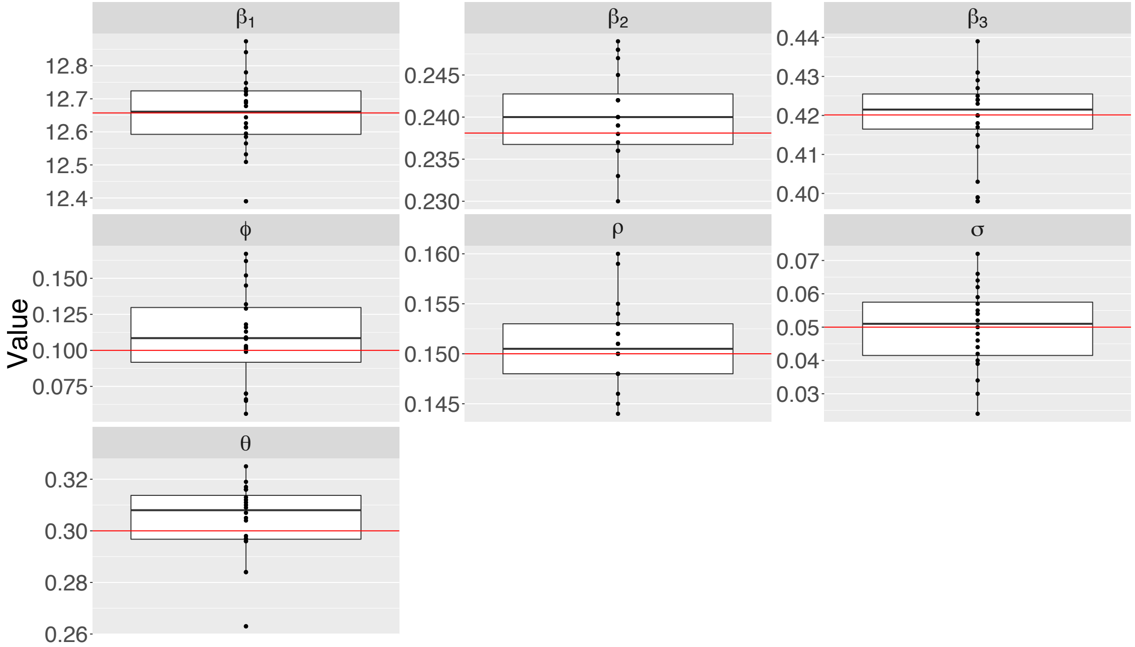

Fig. D.3. Plot of the MLEs inferred from 20 model realizations of Model St+St+. The red horizontal lines are the true parameter values used to generate the simulations.

D.1 *Robustness study*

The robustness study as described in Section 5 of the main manuscript was carried out with 2000 particles and 200 iterations for the modes with stochastic transmission models (StSt+, St+St+, St+St, StSt) and all other algorithm specifications as described in Section B. We report the AIC values for each model fit in Table D.3. We see clearly that for each realization, the respective true model always has the lowest AIC which confirms the correctness of our methods (i.e. best fitting model is always on the diagonal). Figure D.4 gives an example of the incapability of Model St+St to fit data under model misspecification.

	DtSt	DtSt+	StSt+	St+St+	St+St	StSt
DtSt	**13132.58**	13134.57	13615.32	13613.36	13609.48	13606.16
DtSt+	2487951.13	**21248.82**	21250.20	21252.04	31886.44	32088.62
StSt+	4428816.06	22650.99	**22632.64**	22634.34	32087.86	32256.70
St+St+	3178656.57	22285.19	22233.86	**22217.30**	31675.86	32083.00
St+St	355054.81	-	-	-	**16163.90**	23121.36
StSt	140607.80	-	-	-	-	**14912.24**

Table D.3. AIC values from the robustness study. The rows denote from which model the data set was generated and the columns denote to which model the respective simulated data set was fitted to. In bold the minimum AIC value of each row. No entry indicates that a substantial amount of filtering failures occurred so no reliable search was carried out.

D.2 *Simulation study AIC*

We carried out a small simulation study in order to investigate if the AIC can detect potential misspecifications in the transmission model in an endemic SIRS framework when only observing new cases aggregated over some time interval, as is the case for the rotavirus data. As proof of concept we specifically focused on misspecifications due to (1) alternative forms for random elements in the force of infection, (2) beyond-exponential distributed infectious period and (3) seasonality. We only report our findings in the following, a detailed description of the set up of the study and the code can be found in Stocks (2017). (1) The 6 models in our manuscript differ with respect to the amount of stochasticity in the transmission model (especially in the

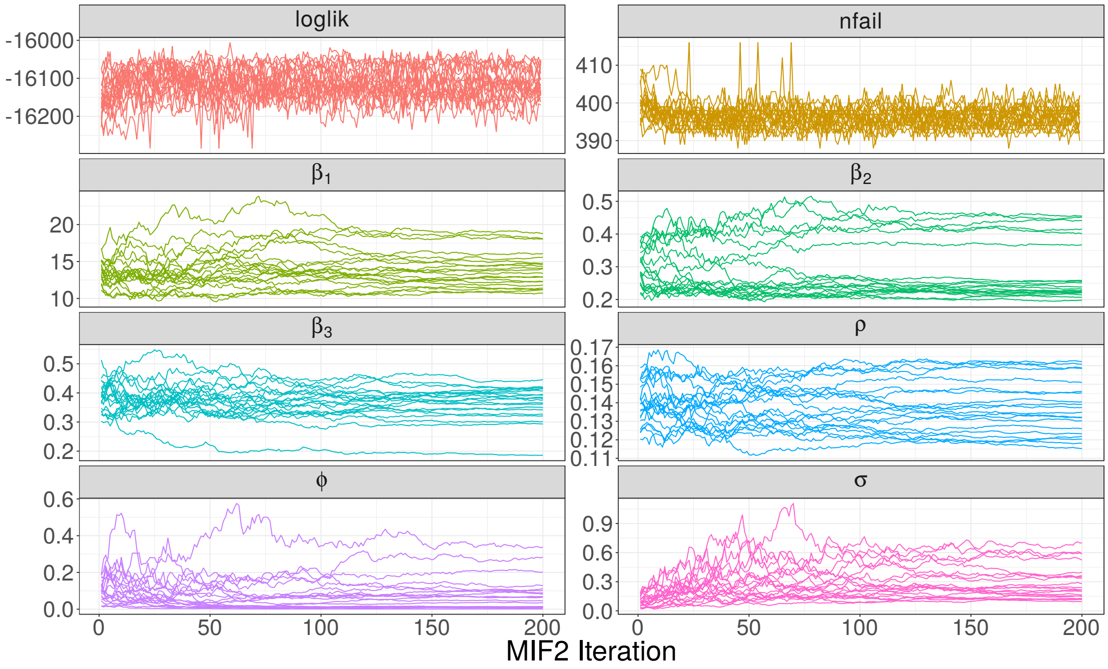

Fig. D.4. Diagnostic plot of the iterated filtering algorithm fitting Model St+St to a model realization from Model StSt+. Shown is the evolution of the log-likelihood, the number of filtering failures (nfail) and parameter estimates per iteration for 20 trajectories with random starting values drawn from a hypercube. The number of filtering failures in each iteration is very high and does not decrease as iterations proceed. Hence, a reliable parameter search can not be carried out. We observe the same phenomenon for Model StSt (not shown here) which is why we excluded both models from our analysis.

force of infection) and the amount of overdispersion in the observation model. Here we definitely see some merit in the AIC approach as it in each case identifies the correct model as the one with lowest AIC, cf. Table D.3. (2) In a simple SIRS model with birth and death in an endemic state scenario as the one of our rotavirus modelling we tried to see, if identification of a gamma distributed infectious period would be detectable through AIC. We found that in our study the AIC chooses the right model in 90% of the cases if the data is fitted to a model with a gamma distributed infectious period but only in 40% cases if the data is fitted to the model where the infectious period is exponentially distributed. (3) We simulated from the simple SIRS model described above and a SIRS model with seasonality. We find that the AIC clearly picks the right model for each of our 10 simulations. We conclude that the AIC for epidemic models in endemic

state can explicitly help to discriminate between forms that directly affect the transmission event. Through our small simulation study in the context of a SIRS model, we showed this for forms of seasonality and additional stochasticity in the transmission model. Non-exponential distributions are harder to be detected as the signal in the data is low, which supports our modelling choice of the simplest version (exponential times) for our model.

E. Model fit rotavirus data

Figures E.5, E.6, E.7 and E.8 show how Models DtSt, DtSt+, StSt+ and Model 18 from Weidemann *and others* (2013) fit the rotavirus data. Although the data clearly lies in the 95% prediction interval for all models with overdispersion in the observation model, we noticed that the model mean for the young children in these models is slightly lower than the data for the first three years of our investigations. This does not occur for Poisson distributed observations so we initially presumed that the phenomenon might be an artifact of the negative binomial distribution. We carried out some additional analysis in order to understand this better. Firstly, we introduced age-dependent overdispersion parameters which improved the log-likelihood, however, did not raise the mean in the first age group compartment. Secondly, we assumed observations to be drawn from a left-censored normal distribution with the same mean and variance as for the negative binomial distribution in order to avoid the skewness of the negative binomial distribution. Also this approach did not raise the mean in the first age group so we conclude that the observed underestimation is not due to the negative binomial distribution but rather a general consequence of having overdipsersion in the model.

For the comparison with Weidemann *and others* (2013), we solved the ODE system of Model 18 for the posterior mode of the fitted parameters and from this solution calculated the expected number of cases in each age group, respectively. The log-likelihood is then obtained by summing over the negative binomial density evaluated at the data for each time step. In order to calculate the AIC, we chose the number of parameters that were estimated, i.e. 17 for Model 18, which makes it a conservative estimate since the parameters were calculated with an a prior distribution. Moreover, Model 18 was originally fitted assuming a linear increase of the reporting rate in the year 2004 and which was model specific – we, however, scaled up the original data by the averaged posterior density using the weights from the vertical averaging regarding contact pattern C6 and did not use linear increase in year 2004 but a step function.

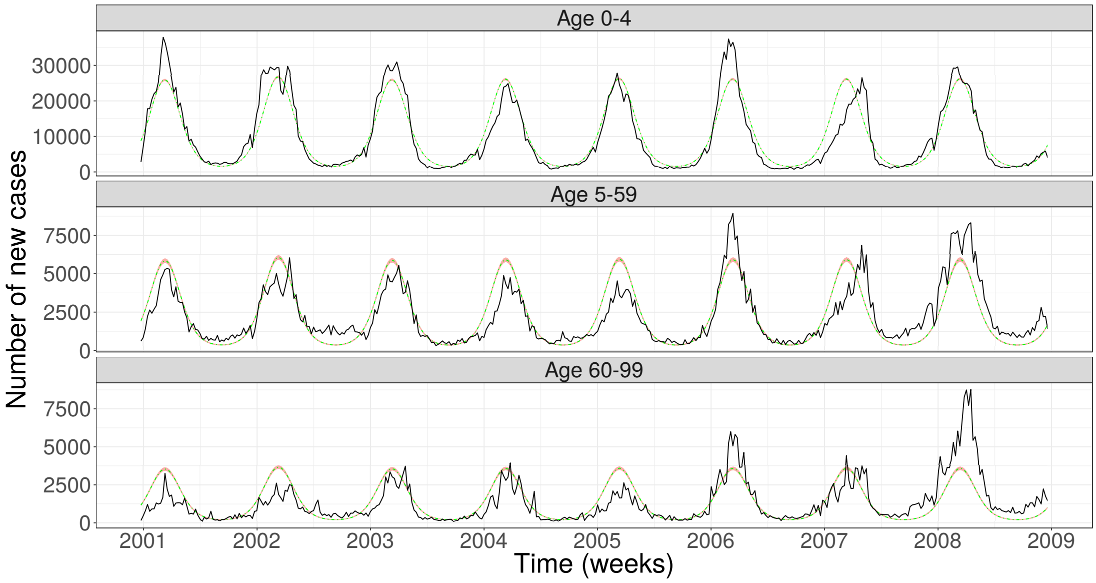

Fig. E.5. Model fit of Model DtSt to rotavirus incidence data (solid back line) and the model mean (solid light line). The 95% prediction interval is very thin so it is nearly indistinguishable from the model mean.

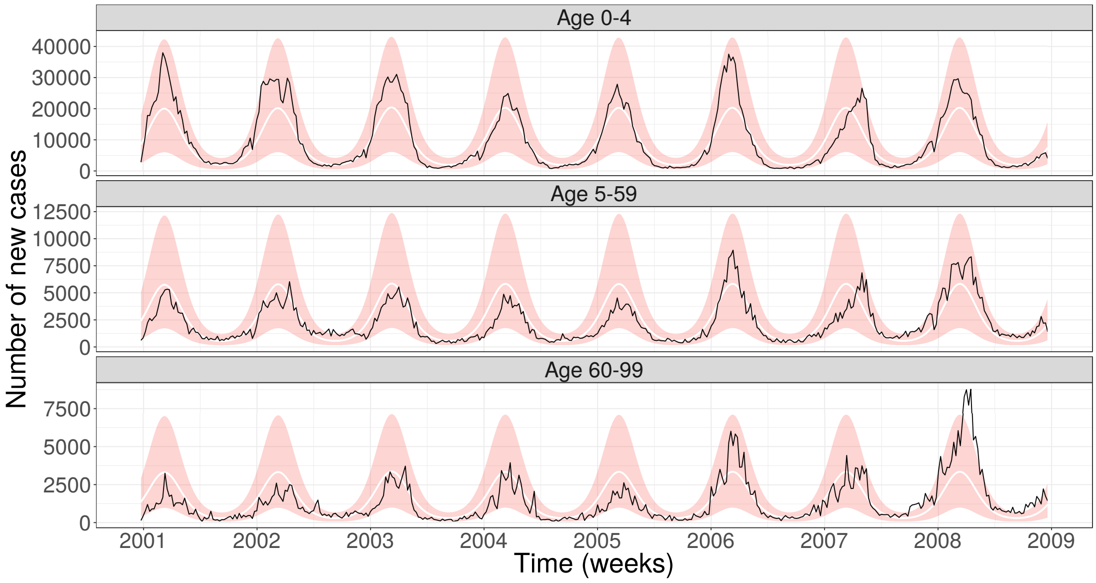

Fig. E.6. The 95% prediction interval (shading) for realizations of Model DtSt+ evaluated at the maximum likelihood estimate for the rotavirus incidence data (solid back line) and model mean (solid white line).

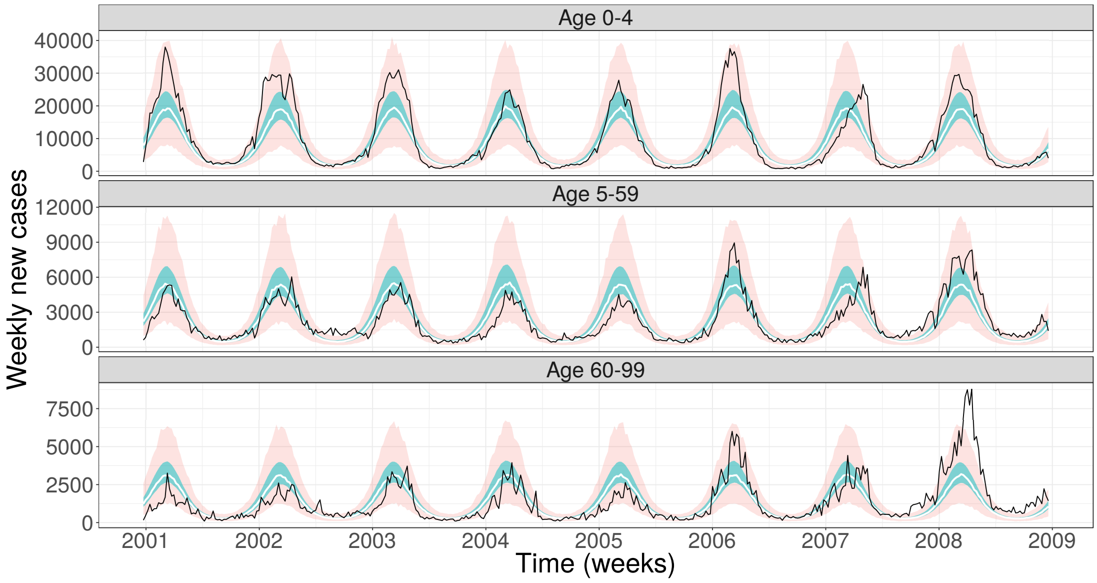

Fig. E.7. The 95% prediction interval (light shading) for 1000 realizations of Model StSt+ evaluated at the maximum likelihood estimate for the rotavirus incidence data (solid back line) and the median (solid white line). Furthermore, the 95 % prediction interval of these 1000 realizations for only the transmission model is shown (darker shading).

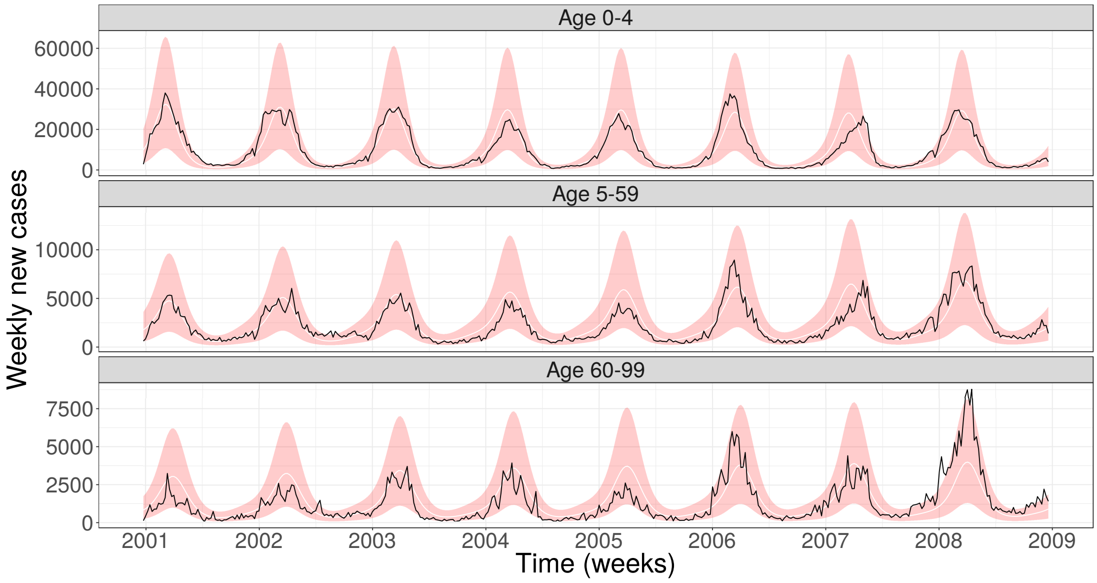

Fig. E.8. The 95% prediction interval (shading) for Model 18 in Weidemann *and others* (2013) assuming no under-reporting, the model mean (solid white line) and the rotavirus incidence data (solid black line). For all computations we used the same dispersion parameter as in Weidemann *and others* (2013) which is a fair approximation as long as the number of cases is fairly large.

E.1 *Diagnostic plots*

Figures E.9 and E.11 show the evolution of the log-likelihood, number of filtering failures and parameter estimates per `mif2` iteration. In the diagnostic plot for Model StSt+ (Figure E.9) one can observe that after the log-likelihood of the perturbed model has increased significantly there is a small drop of the likelihood before stabilizing at a value which seems not optimal after having seen higher log-likelihood values at earlier iterations. However, re-running the particle filter at each iteration returned by the `mif2` model gives that the log-likelihood of the model of interest is increasing before stabilizing, cf. Figure E.10. One possible explanation for the observed phenomenon is that the `mif2` model of Model StSt+ which includes extra variability in the parameters via random-walk perturbations explains the data better if the perturbations are larger. This indicates that the actual target model i.e. the one without perturbations, is not variable enough to explain the data well.

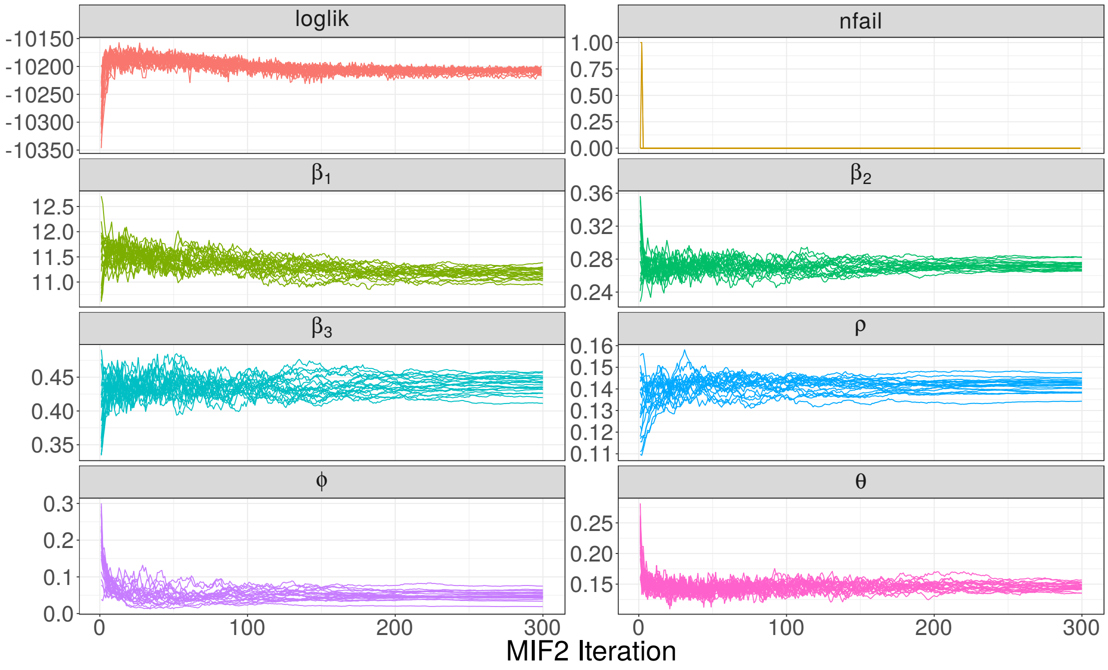

Fig. E.9. Diagnostic plot of the iterated filtering algorithm for Model StSt+for the rotavirus incidence data. Shown is the evolution of the log-likelihood, the number of filtering failures (nfail) and parameter estimates per `mif2` iteration for 20 trajectories with random starting values drawn from a hypercube.

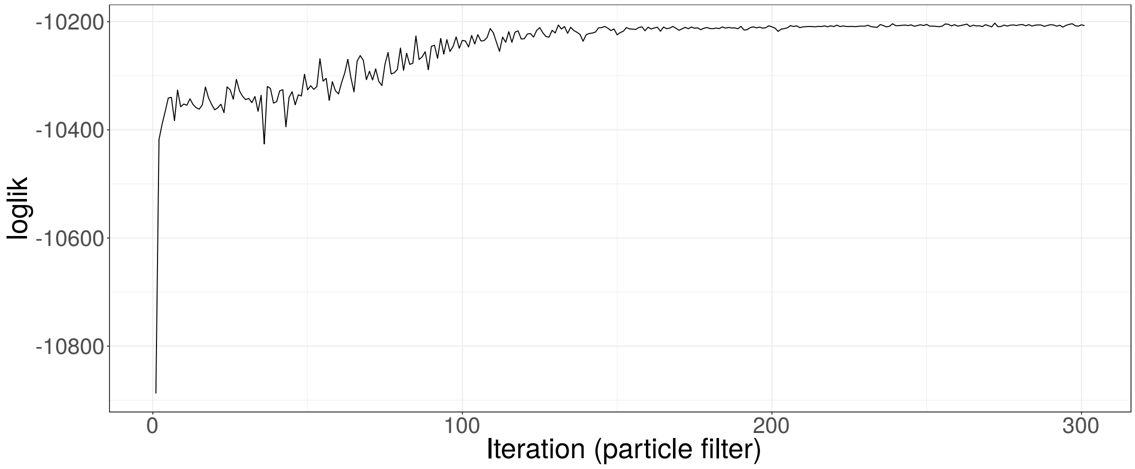

Fig. E.10. Particle filter evaluation for each iteration of one `mif2` run from Model StSt+ (Figure E.9).

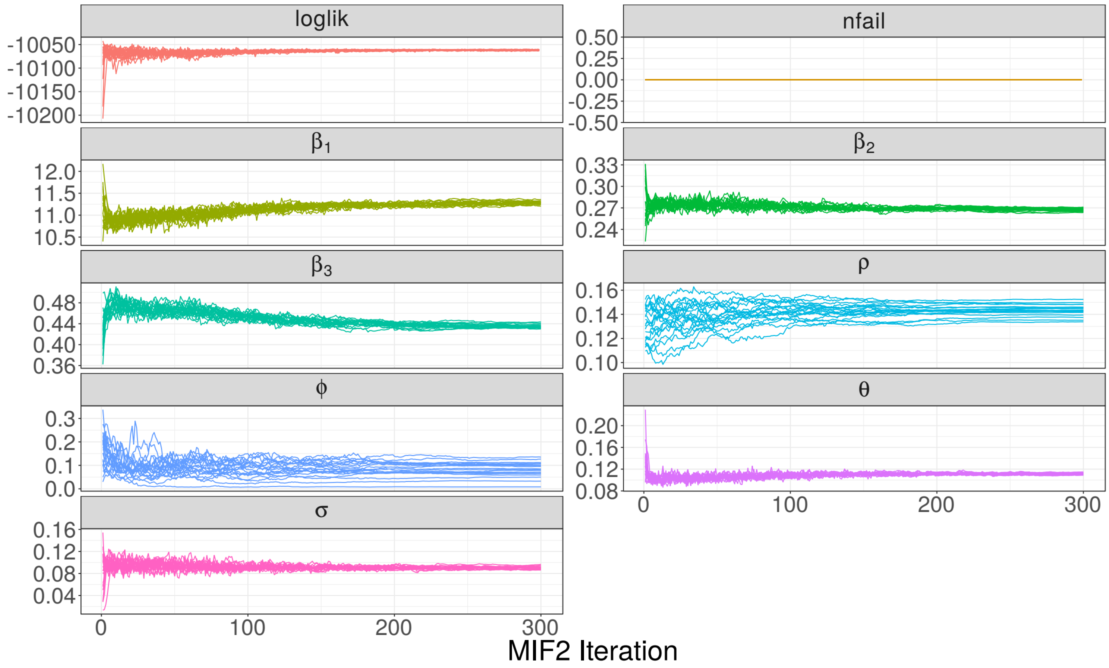

Fig. E.11. Diagnostic plot of the iterated filtering algorithm for Model St+St+ for the rotavirus incidence data. Shown is the evolution of the log-likelihood, the number of filtering failures (nfail) and parameter estimates per iteration for 20 trajectories with random starting values drawn from a hypercube.

E.2 *Discussion on R_0*

The estimates for β_k support that children under the age of 5 years are highly susceptible to the disease while older individuals have much lower susceptibility which slightly rises again later in life. One could argue that the value of R_0 is surprisingly close to the value one for a highly infectious disease as rotavirus. For this, it should be noted that the age-specific basic reproduction number for children under 5 years of age is very high ($R_0^{(1)} = 11.26$ for Model St+St+), but this age class in our model only makes up approximately 6% of the total population. The age-specific basic reproduction numbers of the two older age groups are much lower ($R_0^{(2)} = 0.27$ and $R_0^{(3)} = 0.43$ for Model St+St+). This might have different reasons: firstly, young children might truly have a higher susceptibility to the disease than older individuals. Secondly, it is possible that young children mix at a higher rate and expose themselves more to the disease by close body contact. Another possible scenario is that with higher age there might be partial immunity from earlier infections left which is why older individuals do not get ill. Furthermore, the severity of symptoms could play an important role for the reporting behavior of the disease: it might be that symptoms are very severe in younger children which lead to a higher reporting rate, while cases in older individuals are less symptomatic or even asymptomatic and hence are not reported. Further, even if symptoms are the same in all age classes, older individuals might not consult the doctor as often. Both of the last two explanations we would not be able to detect, because we assumed the same scaling rate for the under-reporting and fixed the rate of waning of immunity, ω, for all age classes. A sensitivity analysis for ω indicated that the longer the time of natural immunity lasts, the higher is R_0. For example, if we choose the time until natural immunity 50 years, the basic reproduction rate increases to 1.44. Moreover, R_0 depends heavily on the reporting rate we used to scale up the data – if the true under-reporting rate is lower than we assumed, R_0 increases. Of course we would have liked to disentangle all the previously mentioned explanations from each other, but the important message is that without any additional structural insights,

the available data is too coarse to do that.

References

Gillespie, D. T. (2001). Approximate accelatered stochastic simulation of chemically reacting systems. *Journal of Chemical Physics* **115**(4), 1716–1733.

Keeling, M. J. and Rohani, P. (2008). *Modeling Infectious Diseases in Humans and Animals*. Princeton University Press.

King, A. A. and Ionides, E. L. (2017). Project pomp on github. *http://kingaa.github.io/pomp/* [**Accessed September 15, 2017**].

Nelder, J. A. and Mead, R. (1965). A simplex method for function minimization. *The Computer Journal* **7**(4), 308–313.

Stocks, T. (2017). Project pomp-astic on github. *https://github.com/theresasophia/pomp-astic* [**Accessed October 2, 2017**].

Weidemann, F., Dehnert, M., Koch, J., Wichmann, O. and Höhle, M. (2013). Bayesian parameter inference for dynamic infectious disease modeling: rotavirus in Germany. *Stat Med* **33**(9), 1580–1599.

[*Received October 16, 2017; Revised May 13, 2018*]
